# Biodegradation kinetics and interactions of styrene and ethylbenzene as single and dual substrates for a mixed bacterial culture

**DOI:** 10.1186/s40201-015-0230-y

**Published:** 2015-10-19

**Authors:** Hossein Hazrati, Jalal Shayegan, Seyed Mojtaba Seyedi

**Affiliations:** Department of Chemical and Petroleum Engineering, Sharif University of Technology, Tehran, Iran

**Keywords:** Styrene, Ethylbenzene, Mixed culture, Andrews model, SKIP model

## Abstract

This study examined biodegradation kinetics of styrene and ethylbenzene as representatives of alkenylbenzenes and mono-alkylbenzenes, respectively. The compounds were studied independently and as binary mixtures using a series of aerobic batch degradation experiments introduced by acclimatized mix culture. Initial concentration of styrene and ethylbenzene in the liquid phase vacillated from 0 to 220 mg/l. The Andrew model was applied for the biodegradation of individual substrates and the estimated constants of the equation for styrene and ethylbenzene were μ_max_ = 0.1581, 0.2090 (1/h), K_S_ =25.91, 37.77 (mg/L), K_I_ =13.15, 62.62 (mg/L), respectively. The accomplished parameters from single substrate degradation tests were used to predict possible interaction factors achieved from dual substrate experiments. The Sum Kinetics with Interaction Parameters (SKIP) model and the purely competitive enzyme kinetics model were employed to evaluate any interactions. The SKIP model was found to accurately describe these interactions. Moreover, it was revealed that ethylbenzene plays an influential role on styrene consumption (e.g. I_E,S_ = 1.64) compared to styrene which has insignificant inhibitory effect on ethylbenzene usage (e.g. I_S,E_ =0.4) . The active site differences for styrene and ethylbenzene biodegradation and the pathway variations for biodegradation are among the major potential reasons for failure of the estimation that occurred in purely competitive kinetics model. This study is the first to calculate the interactions between styrene and ethylbenzene.

## Introduction

Nowadays, the elimination of Volatile Organic Compounds (VOCs) from contaminated airstreams and groundwater has become one of the main issues facing the industrialized world [1].

Various methods exist for the treatment of the wastewaters containing VOCs such as physical (e.g. adsorption with the activated carbon) or chemical processes (e.g. Advanced Oxidation Processes). Meanwhile, the biological treatments are gradually becoming popular due to the complete destruction of contaminants [2, 3]. Compared to the physical and chemical methods, biological processes have excessive green benefits and are potentially cost saving either for capital or operating costs. These benefits made biological processes appropriate for the treatment of wastewaters containing various organic pollutants [4]. Two main VOCs that are widely used in many petrochemical and polymer-processing industries are styrene and ethylbenzene. In the chemical industry, styrene monomer plays a pivotal role and it is mainly used in the production of polystyrene and several copolymers [5]. This significant component is produced in large scales through dehydrogenation of ethylbenzene. Despite various advantages, the low conversion rate of styrene and ethylbenzene are considered as residues present in most solvents [6]. These VOCs are known to be hazardous to human health and the environment. The negative effects of their short-term and long-term exposure to human health and environment have been validated in several studies [7, 8]. The Maximum Contaminant Level (MCL) for ethylbenzene and styrene is 0.7 mg/L and 0.1 mg/L, respectively [9]. Hence, to achieve the standard concentration (e.g. below MCL) of styrene and ethylbenzene levels, the liquid and gaseous effluents from petrochemical complexes, polystyrene factories, and plastic industries need to undergo an appropriate treatment before being discharged to the environment.

One of the major steps to forecast and optimize the biodegradation procedures at commercial scales is to specify the degradation kinetics of these contaminants by bacterial populations. Therefore, the employment of an appropriate kinetic model is necessary. For instance, Monod derived models are employed for population growth studies during the microbial growth kinetics [10]. The Monod kinetic model is commonly employed in previous studies where there were pure culture, restricted substrate, and non-inhibitory biomass growth [11, 12]. However, the modified Monod models have been used to investigate the effects of substrate inhibition on biomass growth at large quantity of substrates [13–17].

The styrene biodegradation kinetics have been previously studied and modeled for special isolated microorganisms as well as mixed bacterial cultures. The fungus *E. oligosperma* [18] and some bacteria such as *Rhodococcus pyridinovorans PYJ-1* [19] constitute small part of the microorganisms which are able to treat synthetic wastewater and gases containing styrene. R. Babaee et al. [16] used the industrial activated sludge as a mixed culture to biodegrade styrene in synthetic wastewater. The authors have been successfully modeled and fit the Andrews kinetics to their data [16]. Ethylbenzene biodegradation kinetics were modeled either by special isolated microorganisms or through the mixed bacterial culture as a sole source of carbon and energy. Bacterial strains, including *Pseudomonas fluorescens-CS2* [20], several strains of *Pseudomonas putida* [21], and *Alcaligenes xylosoxidans* [22] were used to biodegrade ethylbenzene. Another research study by H. Shim et al. [23] also evaluated the biodegradation of ethylbenzene in co-culture. The study concluded that there is a direct relationship among biodegradation rates of BTEX, BTEX concentration, and the reactor loading rate [23].

The results from previous studies indicate that other substituents of the mixture can be intensely affected by the microbial degradation of a compound [24]. Such interactions can involve the enhancement (positive effect) or inhibition (negative effect) of degradation of substrates in mixtures. However, negative interactions are reported more frequently [25, 26]. This indicates that taking the metabolic influence of each compound into account is vital for further understanding of the mixture effects in microorganisms.

The analytical outcomes assist to develop the applications of the biological systems for efficient VOCs degradation which enhances the energy savings. In addition, there are limited number of studies which model the styrene and ethylbenzene biodegradation mixtures in water. Although in the petrochemical industry these materials coexist with each other, the interaction between the two substrates (styrene and ethylbenzene as a binary mixture) and the development of the mixed culture in this circumstance have not been modeled to date.

Overall, this study aims to determine the biodegradation kinetic constants under well-defined conditions in the laboratory and quantify the interactions that emerge during the degradation of styrene and ethylbenzene by making use of the interaction equations. It also endeavors to devise a method to calculate the approximate value of biomass growing for a bacterial group.

## Materials and methods

### Assessment of model adequacy and parameters

Due to suitable and long-term maintenance of the cultures under strong substrate, this study chose the batch cultures to attain biodegradation kinetics, to estimate models, and to define model parameters. Biomass growing can be defined by Eq. (1) in batch situation. This equation is suitable for biomass growth due to different number of substrates [27].

1 $$ \frac{\mathrm{dX}}{\mathrm{dt}}=\mu X $$

For a low volatility chemical, in a batch degradation for a given substrate, *i*, the substrate depletion equation was:

2 $$ \frac{\mathrm{d}{S}_i}{\mathrm{d}\mathrm{t}}=-\frac{\mu_iX}{Y_{\raisebox{1ex}{$X$}\!\left/ \!\raisebox{-1ex}{${s}_i$}\right.}} $$

In these expressions, X is biomass concentration (mg/l), t is time (h), *μ*_(*i*)_ is specific growth rate (h^−1^), *S*_*i*_ is substrate concentration (mg/l), and ${\mathrm{Y}}_{\mathrm{X}/{S}_i}$ is the observed yield coefficient (mg/mg), defined as the proportion of the biomass mass generated to the mass of substrate consumed.

The specific growth rate in Eq. (1) and Eq. (2) could be described by various models. Monod initially suggested the idea that the microbial growth kinetics has been controlled by an empirical model (Eq. 3) [28].

3 $$ {\mu}_i = \frac{\mu_{\max_{i\ }}{S}_i}{{K_s}_i+{S}_i} $$

Where specific growth rate of biomass is ${\mu}_{{}_i}$ (h^−1^), ${\mu}_{ma{x}_i}$ is the maximum specific growth rate of biomass (h^−1^), *S*_*i*_ is substrate concentration (mg/l), and *K*_*si*_ is substrate half-saturation constant (i.e. substrate concentration at half ${\mu}_{ma{x}_i}$).

A modified version of the Monod model is hired to deliver an improved fit for the achieved data from the sole substrate tests. In this case, Monod derivative (e.g. the Andrews model) shown as Eq. (4) was used for substrate inhibition [28].

4 $$ {\mu}_i = \frac{\mu_{\max_{i\ }}{S}_i}{{K_s}_i+{S}_i+\raisebox{1ex}{${S}_i^2$}\!\left/ \!\raisebox{-1ex}{${K}_I$}\right.} $$

In Eq. (4), ${\mu}_{ma{x}_i}$ is the maximum specific growth rate (h^−1^), *K*_*si*_ is the half velocity constant, and *K*_*I*_ is the substrate inhibition constant which quantifies the influence of a toxic compound on its biodegradation. It is important to note that the Andrews model considered as a nonlinear equation. Thus, it is hard to reliably estimate parameters such as linier equations. To overcome this barrier, nonlinear least-squares regression was hired to minimize possible errors during the prediction of mentioned kinetic parameters.

The growing number of substrates led to further complexity of degradation models. Moreover, kinetic parameters for a single substrate are not able to describe the phenomena observed during the degradation of mixtures. Uncompetitive inhibition, non-competitive inhibition and competitive inhibition are some interactions that can take place once multiple substrates are present. One of the most common types of these models is obtained through the summation of specific growth rates on each substrate. In this environment, a sum kinetics model which incorporates purely competitive substrate kinetics could be useful (Eq. 5) [29].

5 $$ {\mu}_{tot}={\mu}_1+{\mu}_2 = \frac{\mu_{\max_{1\ }}{S}_1}{{K_s}_1+{S}_1+\left(\frac{{K_s}_1}{{K_s}_2}\right){S}_2}+\frac{\mu_{\max_{2\ }}{S}_2}{{K_s}_2+{S}_2+\left(\frac{{K_s}_2}{{K_s}_1}\right){S}_1} $$

Nevertheless, when the ways of interactions among substrates are not thoroughly necessary, use of model which deal with different interactions deprived of their specifics might be the best choice. This model is known as Sum Kinetics with Interaction Parameters (SKIP) and formulated by incorporating I_i,j_ as an unidentified interaction factor.

6 $$ {\mu}_{tot}={\mu}_1+{\mu}_2 = \frac{\mu_{\max_{1\ }}{S}_1}{{K_s}_1+{S}_1+{I}_{2,1}{S}_2}+\frac{\mu_{\max_{2\ }}{S}_2}{{K_s}_2+{S}_2+{I}_{1,2}{S}_1} $$

In Eq. 6, I_i,j_ specifies the degree of impact which the “i” substrate enforces to the “j” substrate biodegradation. According to the model as the amount inhibitory increases, the I_i,j_ (interaction factor) enhances gradually [26]. The value of I_i,j_ is calculated by fitting the SKIP model to a dual of mixture of containments. This is performed by defining some basic parameters related to each substrate such as μ_m_, K_s_, and Y_X/S_ and replacing them in the cited equation.

### Culture and media

A suitable culture with a reliable background plays significant role to properly evaluate the kinetic parameters on growth and substrate removal of any compound [30]. In this study, the industrial mix culture is supplied from a petrochemical complex^1^ located in Tabriz (North Western Iran). Tabriz petrochemical complex is a producer of raw polymers such as polystyrene as well as consumed raw materials such as styrene and ethylbenzene. In order to improve the capability of the bacteria and to modify their macromolecular composition (e.g. protein, RNA, and DNA) in response to their environment, the bacterium culture was grown under aeration in the synthetic wastewater. Table 1, shows the components of the carbon-free growth medium formulation. All nutrients used in the growth medium were obtained from Merck Ltd. The mineral media was supplemented with styrene and the ethylbenzene were obtained from Merck Ltd. The concentration ranges of styrene varied from 0 to 220 mg/l while ethylbenzene ranged from 0 to 220 mg/l in single substrate experimental. Both nutrients as carbon sources were equivalent to a COD of 675 ± 10 mg/l and were prepared in test flasks. In dual substrates tests various concentration of styrene and ethylbenzene were used as a binary mixture to set a COD equivalent to 200 ± 10.

**Table 1:** Mineral concentrations in the bioreactor at the beginning and the end of the acclimation step

Constituent	Concentration (mg/L)
NH_4_Cl	560
K2HPO4	35
KH_2_PO_4_	45
MgSO_4_.7H_2_O	13
CaCl_2_.2H_2_O	7
FeCl_3_	5
ZnSO_4_	2
NaHCO_3_	500
EDTA (C_10_H_16_N_2_O_8_)	7

Since ammonia was employed as a nitrogen source in biomass cells during the production of main elements, Ammonium chloride (NH_4_Cl) was used to supply nitrogen for the medium. The phosphate salts were added to the synthetic medium to provide a buffer capacity and to acts as a source of phosphorus for the microorganisms. EDTA was used in low level of concentration as a chelating agent.

### Experiments

To distinctively evaluate the values of each parameter and identify the parameters correctly, kinetic experiments need to be performed in a condition with minimum error levels. Therefore, instabilities must be small and/or the test session that leads to the kinetic measurements needs to be short. In addition, another element that intensely impact the estimated value of each parameter in kinetic experiments is the ratio of the initial substrate concentration, S_0_, to the initial biomass concentration, X_0_, [30]. In our experiments, since inflexibility exists during continuous tests (as mentioned previously in this report), batch kinetic experiments were carried out in 250-mL amber-colored serum bottles. Separate tests were performed for styrene and/or ethylbenzene and the biodegradation of these compounds were examined individually and together.

The first set of experiments involved the use of sole substrate (e.g. styrene or ethylbenzene, separately) to become biodegraded by the mix culture. Therefore, kinetics experiments on nine original concentrations of substrate, from 8 up to 220 mg/L which is the maximum dosage of petrochemical plant (8, 21, 28, 37, 60, 80, 103, 122, 162, 220 mg/L for styrene as well as 12, 23, 32, 42, 64, 83, 98, 130, 158, 220 mg/L for ethylbenzene). Concurrently, the early concentration of biomass for the all bottles is kept static at 25 mg/L. Besides, the biomass concentration has been experimentally observed over time in order to obtain the specific growth rate and to combine this parameter into a model. The concentration of biomass and substrate at different time pauses were detected using the technique cited in the following section (e.g. Section 2.4). The gas phase was also observed randomly during the experiments. 25 mg/L of the biomass in addition to the 100 mL mineral medium were in 250 mL sample bottles (sterile amber-colored serum bottles sealed with Teflon-coated silicone septa and Paraffin layer to prevent volatilization) in the rotary incubator shaker at 160 rpm. Besides, the experiments in which styrene and ethylbenzene were simultaneously biodegraded as mixed substrates, the initial biomass concentration range was 8–12 mg/L. In fact, the initial substrate concentration to the initial biomass concentration ratio ranged from 22.0 to 27.5 on COD basis. This range tolerates determination of the inherent growth related kinetic factors. It characterizes the abilities of the members who belong to the activated sludge with the rapid growth kinetics [30]. In addition, the biomass quantities were selected to diminish the possible errors caused by leaks, to reduce the required time for complete biodegradation, and to eliminate any changes in the characteristics of the biomass caused by long-term contact to the VOCs or some probable by products. The temperature and pH of the aqueous solution were kept stable at 32 °C and 7, correspondingly, until the tests were finalized. It is worth noting that all batch experiments were achieved by concurrent incorporation of similar batches which are free from substrates.

### Analytical methods

The GC (Agilent 6890) was set with a Flame Ionizing Detector (FID) and attached to a silica HP-Innowax capillary column (30 m × 0.32 mm × 0.5 lm, J&W Scientific, USA) that was aimed for an appropriate analysis of volatile elements. High-purity helium poured through the column at 1.5 ml/min and 45 psi as a carrier gas. The injector and detector temperatures were fixed at 220 °C and 280 °C. The initial temperature was programmed at 60 °C for 6 min long and it remained constant for 10 min after it was increased to 150 °C at heating rate of 30 °C/min. Once several test time intervals are given, a 25 μL gas-tight syringe employed to extract suitable amount of gas (10 μL) from each serum bottles. The achieved outcome from the gas chromatograph device was documented on a computer fitted out with Agilent data analysis chemstation^2^ software to execute peak integration and the related exploration. The achieved output was also compared with the calibration curves of individual components and consequently the VOC concentrations were attained. In order to assess the concentration of carbon source(s) (styrene, ethylbenzene) in the aqueous solutions, partition coefficient of carbon source(s) as well as other equations accosiated with Vapor-Laquid Equilibrium (VLE) was hired. To gain the amount of partition coefficient for each components, specific quantity of styrene and ethylbenzene (0.2, 0.5, 0.75, 2.5, 4.5, and 5 μL) were added independently to 250 mL amber-colored serum bottles holding 100 mL mineral medium. Moreover, to avoid any volatilization of styrene and ethylbenzene, the bottles were sealed by Teflon-coated silicone septa and with Paraffin. Once the proper time was allowed and the VLE circumestance is attaind, the styrene or ethylbenzene concentration in the gas phase was evaluated with GC. The partition coefficient of styrene and ethylbenzene was calculated using the mathematical relations and considering the entire volume of VOCs added to serum bottle [19]. To estimate the biomass concentration in the liquid medium the cell concentration suspending in the liquid was examined. The results from fresh culture medium were used as an index in order to compare and evaluate the obtained results from samples with various amount of biomasses. The Optical Density (OD) was determined at 600 nm using a spectrophotometer (Spectro Direct 712000; Lovibond®, Tintometer® Ltd, England). The output was plotted in a graph as a standard curve for desiccated mass of biomass per volume, mg/l, against different quantities of the ODs’ resulted at 600 nm. As a consequence, with the acceptable total regression (R^2^ = 0.9979) a linear curve was established up to concentration of 250 mg/l. To discover other crucial factors such as TSS, VSS, MLSS, MLVSS, and the COD analysis were accomplished according to the standard methods [31]. During the tests time the pH value was fixed and measured by a pH-meter (SCHOTT® CG825). All quantities were prepared using the same method if necessary.

## Results and discussions

### Single substrate experiments and biodegradation kinetics

#### The effect of single substrates concentration

To handle kinetic experiments, a specific amount of styrene and/or ethylbenzene (deeply discuss in section 2.4) were added to the serum bottles via a 10 μL syringe inserted through the Paraffin protective layer into Teflon cap. The concentration of gaseous phase was monitored during incubation until complete degradation of styrene was attained.

**Fig. 1:**
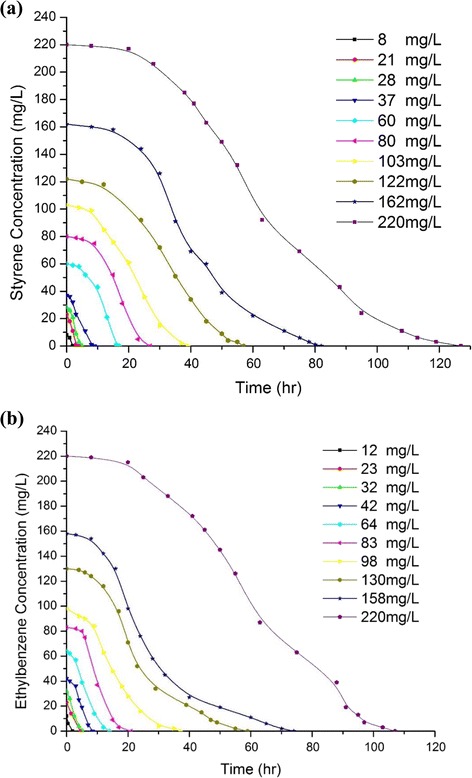
**a** The effect of various initial concentrations of styrene in the liquid phase on degradation period, **b** The effect of different preliminary concentrations of ethylbenzene in the liquid phase on depletion period

Figure 1a and b show the variation of styrene and ethylbenzene concentration in the liquid phase during the batch growths over different time intervals. Various dosages (e.g. 8 up to 220 mg/L for styrene and 12 up to 220 mg/L for ethylbenzene mg/L) were degraded by the microbial community presented in the industrial activated sludge from 2 up to almost 127 h for styrene and from 2 up to 106 h for ethylbenzene. The temperature was fixed at 32 °C and the pH level was 7 for styrene and ethylbenzene. That degradation rate depends on the initial concentration. In addition, inhibition at high concentration diminishes the biodegradation rate [16, 19]. A similar explanation could be drawn from Fig. 1. The figure shows that as the initial concentration grows, the slope of the curve constantly starts to decrease. Moreover, due to the enrichment of inhibition by substrate, the amount of substrate intake declines with the increment of original concentration.

#### The kinetics of biodegradation

Generally, the kinetic model factors are obtained by observing the biomass growth rate over time at different initial substrate concentrations during batch experiments. Consequently, if endogenous decay was abandoned, Eq. 7 in exponential growth phase could be used to calculate the specific growth rate (*μ*_*g*_) values [32].

7 $$ \ln \left(\frac{X}{X_0}\right)={\mu}_g.t $$

Where X_0_ and X indicate the biomass level at the beginning and at time t; (mg/l) respectively. *μ*_*g*_ is the specific growth rate (h^−1^) and t represents the specific time (h). Hence, to calculate the value of the specific growth rate (μ_g_), the biomass concentration has been experimentally observed during the batch experiments with different substrate concentration and fixed initial concentration of biomass.

**Fig. 2:**
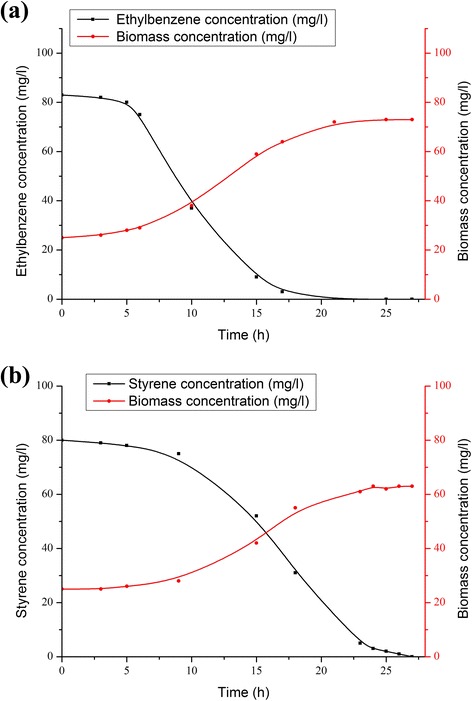
**a** The experimental data for ethylbenzene and biomass concentrations in liquid phase taken from single substrate tests; **b** The experimental data for styrene and biomass concentrations in liquid phase taken from single substrate tests

The kinetics of styrene or ethylbenzene consumption as sole carbon sources by the mixed bacterial cultures which exist in industrial activated sludge is revealed in Fig. 2. The ethylbenzene degradation movements and the parallel biomass progress during the time variation is illustrated clearly in Fig. 2a. The figure specifies that ethylbenzene is fully consumed by nearly 21 h and the sludge growth was imitated by the ethylbenzene consumption. Besides, the sludge growth was apparent after a short lag period (less than 5 h). Unlike the ethylbenzene biodegradation, the data achieved from the styrene biodegradation experiment (Fig. 2b) reveals longer lag period (nearly 9 h) prior to biomass development (Fig. 2a). Also, the styrene consumption was considerably slower (27 h), and similar to the ethylbenzene its growth stopped while styrene concentration was depleted.

#### Styrene

**Fig. 3:**
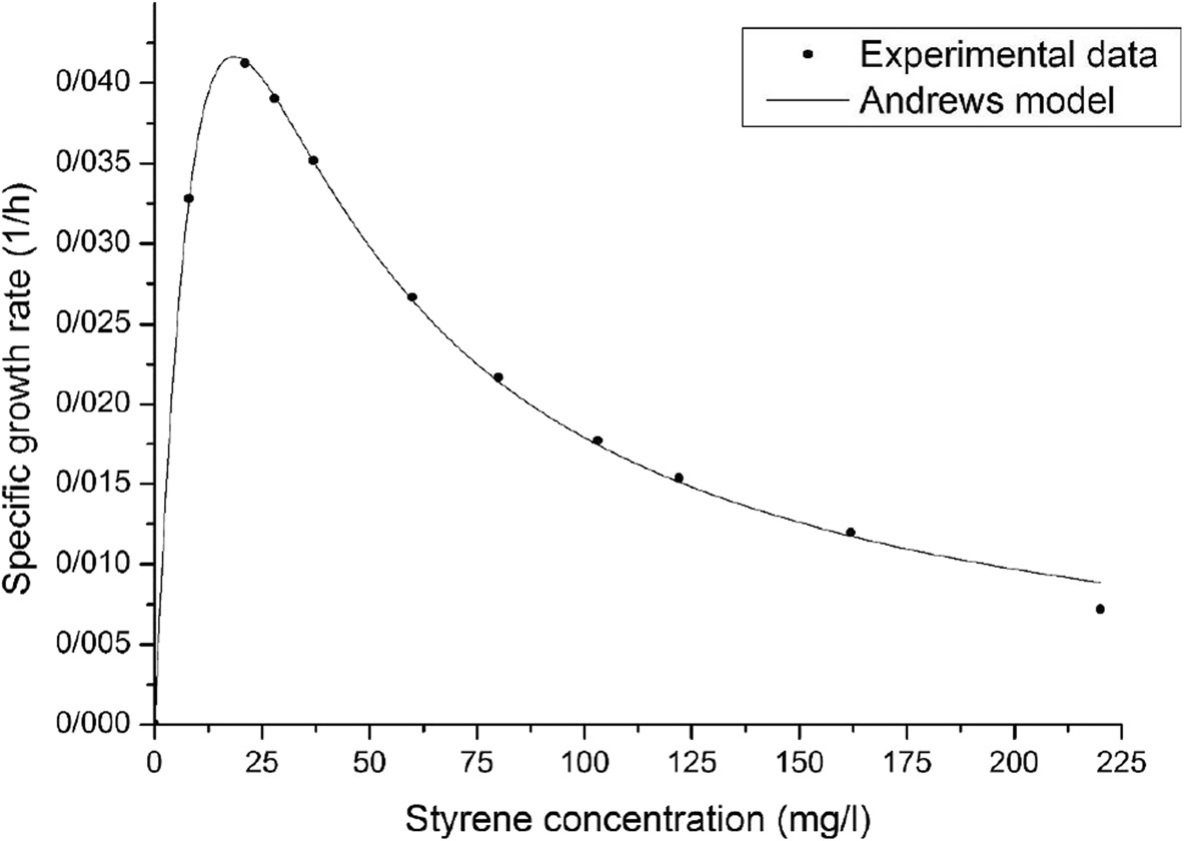
Specific growth rate of the mixed culture at various concentrations of styrene as a sole substrate. Observed experimental data (shapes) and simulation outcomes acquired via Andrews model (lines)

Figure 3 depicts the specific growth rates (μ_g_) plotted against various initial concentrations of styrene as an individual source of carbon. As can be seen, the overall trend shows a similar pattern for the obtained results. The specific growth rate continues to reach the peak value before starting to decline. This indicated the inhibitory effect of substrate above a certain concentration for bacterial activity. Similar to previous researches, trends of graphs and slope of the curves in the second part of plots (after peaks) revealed that as the concentrations of substrates increase, styrene start to make barriers for the bacterial activity [25, 33]. According to the figure, the value of maximum specific growth rates for styrene is 0.042 h^−1^. In addition, the initial concentration of styrene is about 21 mg/l when the peaks achieved in the plots.

The parameters of Andrews model evaluated by curve fitting toolbox provided in Matlab 7.14 software to minimize the least-square error. An acceptable fitness occurs between the parameters of Andrews equation and the experimental data for the mix culture. Details of parameters obtained for styrene are shown in Table 2.

**Table 2:** Estimated parameter values for Andrews kinetic models to biodegrade styrene as a single substrate

Compound	μ_max_ (1/h)	K_S_ (mg/L)	K_I_ (mg/L)	Y_X/S_ (g/g)	R^2^	RMSE
Styrene	0.1581	25.91	13.15	1.19 ± 0.24	0.9917	0.0041

The R^2^ and RMSE parameters approved that the Andrews model has appropriate qualification for our experimental data. Also, former studies of batch operation situations exhibited that the Andrews model has further superior substrate inhibition effect on the cell growth compared to the Monod model when the initial concentration of toxic substrates is increasing (typically S_0_ of beyond 30 mg/L^−1^) [34]. The μ_max_ value shows the capability of microbial culture to use the special pollutant as a source of carbon and energy. As can be seen in Table 3 μ_max_ obtained for styrene is relatively at low level compared to the prior researches that considered the various pure and mix cultures to evaluate the kinetic of biodegradation [35, 36]. This appears to be reasonable enough since the utilized activated sludge constitutes a mixture of several other microorganisms. The abundance of the microorganisms leads to a competition between the bacterium cultures for the common substrate.

**Table 3:** Comparisons between kinetic parameters estimated for the biodegradation of styrene in batch culture in different studies

Strain	Maximum substrate concentration (mg/L)	μ_max_ (1/h)	K_s_ (mg/l)	K_i_ (mg/l)	T (°C)	pH	References
Mix culture adapted with petrochemical residue	220	0.1581	25.91	13.15	32	7	This study
Mix culture adapted with industrial residue	123.4	0.1601	13.8	21.57	32	7	[16]
*exophiala jeanselmei*	104.15	1.26	0.1	3.3	25	5.7	[35]
*P. putida F1*	43	0.86 ± 0.01	13.8 ± 0.9	-------	30	7	[25]
*Pseudomonas sp. E-93486*	90	0.1188	5.984	156.6	30	7	[36]
*exophiala oligosperma*	19.3	0.160	7.381	-------	32.2	5.75	[18]

The parameters’ values are for the **Andrews** model if a value of K_i_ is given. If the K_i_ value is not given the parameters’ values are for the **Monod** model

“K_s_” or the value of half-saturation constant depends on the affinity of a bacterium for a substrate. In other words, bacterium activity with a lower K_s_ value could be further efficient to eliminate the pollutant compared to the upper K_s_ value. According to Table 3, the obtained values for K_s_ are relatively high compared to the previous reports which used pure culture to remove styrene as a substrate [35, 36]. This could be attributed to the activated sludge usage as a bacterial culture as well as to the amount of various microbial species that are probably incapable or poorly accomplished for metabolizing specific carbon sources. This is contrary to the pure culture which included appropriate microbial species to develop growth rate at several concentrations of substrates.

The inhibition coefficient (K_i_), illustrates the impact of component toxicity during treatment process. When bacterial culture has high sensitivity to the component toxic effect, K_i_ quantity is small. However, the large values of K_i_ indicate that the culture is less delicate to substrate inhibition and therefore, the Andrews equation is simplified to the Monod equation. As shown in Table 3, the inhibition coefficients obtained in this study for styrene are fairly high. Thus, it can be concluded that inhibitory effects of styrene is relatively at low level. This is expected since the biodegradation in this study is handled by the mixed culture which covers a wide spectrum of microorganisms and can sustain high tolerances of toxic components compared to the pure microbial culture.

#### Ethylbenzene

**Fig. 4:**
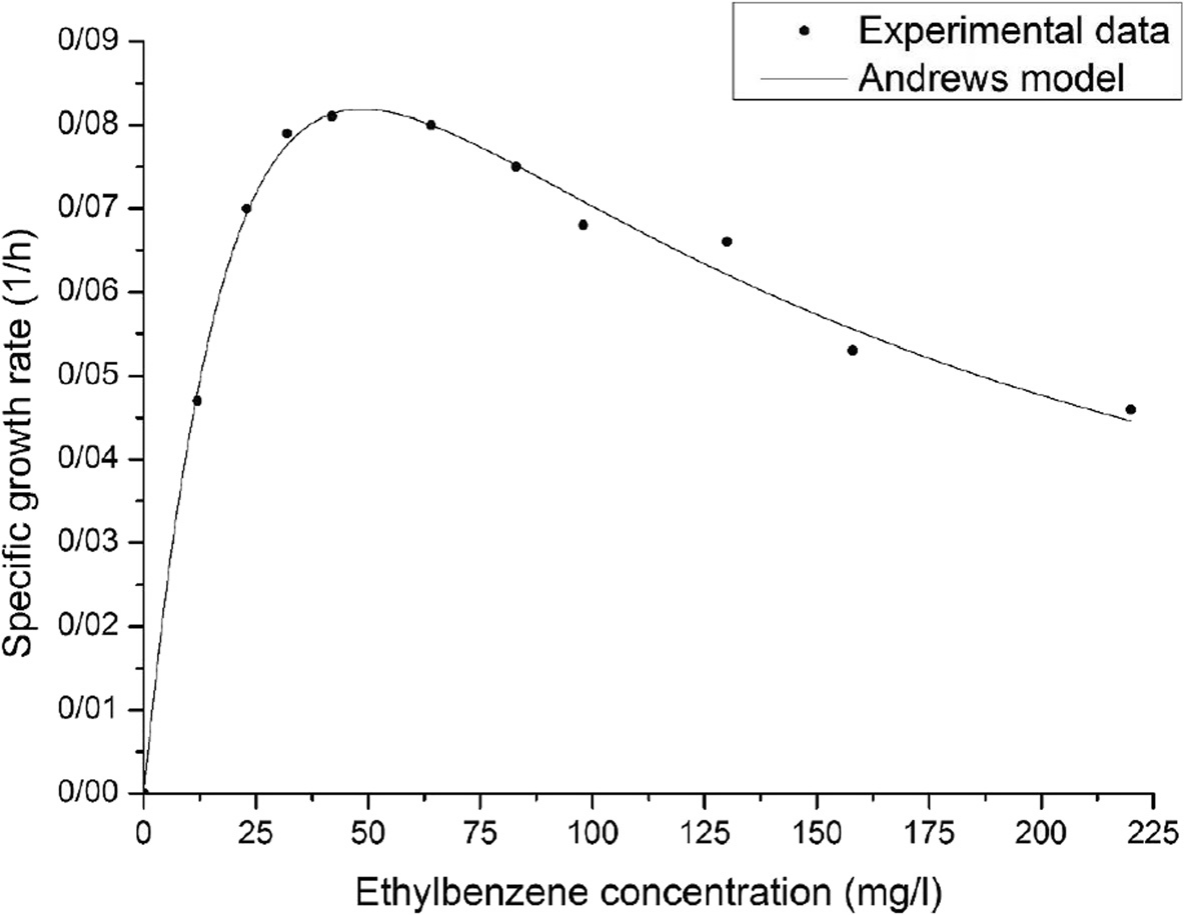
Specific growth rate of the mixed culture at various concentrations of ethylbenzene as a sole substrate. Observed experimental data (shapes) and simulation outcomes acquired via Andrews model (lines)

Data achieved from various initial ethylbenzene concentrations versus specific growth rate (μ_g_) (Fig. 4) reveal that ethylbenzene follows a similar pattern to styrene.

The correlation between the specific growth rate and the initial concentration of the substrate was described by the Andrew kinetic equation with the expected constants (Table 4): μ_max_ (1/h) = 2090, half velocity constant (mg/L) = 37.77, and K_I_ (mg/L) = 62.62. The value of the maximum growth rate for ethylbenzene achieved by the authors is higher than styrene. However, the inhibition coefficient gained for ethylbenzene demonstrates lower toxicity compared to styrene.

**Table 4:** Estimated parameter values for Andrews kinetic models to biodegrade ethylbenzene as a single substrate

Compound	μ_max_ (1/h)	K_S_ (mg/L)	K_I_ (mg/L)	Y_X/S_ (g/g)	R^2^	RMSE
Ethylbenzene	0.2090	37.77	62.62	1.13 ± 0.17	0.9885	0.0030

The R^2^ value for ethylbenzene was 0.988. This demonstrates an acceptable correlation between the experimental and predicted values obtained from the Andrews model.

Table 5 shows the comparison of kinetic parameters for ethylbenzene in bacterial pure and mixed cultures. The maximum specific growth rates, μ_max_, obtained from different Studies was vacillated from 0.006 to 0.26 h^−1^ [36, 37]. The value of μ_max_ in this study also shows the same pattern while the acclimatized mixed culture bacterium shows higher activity of degrading ethylbenzene with μ_max_ of 0.21 h^−1^. The achieved results are close enough to the maximum range reported by Trigueros et al. [36]. Meanwhile, the circumstances in our research with a high amount of substrate concentration created more barriers for microbial culture to biodegrade. Trigueros et al. [36] examined the biodegradation kinetics of ethylbenzene by *R. pyridinovorans PYJ-1*. The specific ethylbenzene degradation rate followed the Andrew model in which S_0_ is the initial ethylbenzene concentration during gas phase. For *pyridinovorans PYJ-1,* μ_max_ (1/h) = 0.26, K_S_ (mg/L) = 1.5, and the inhibition coefficient of 20 mg/L was estimated [36]. The inhibition coefficients K_I_ for ethylbenzene was estimated to be 62.62 mg/L. Compared with previous studies, the relatively high value of K_I_ for ethylbenzene indicated that the mix culture was less sensitive to substrate inhibition [38].

**Table 5:** Comparisons between kinetic parameters estimated for the biodegradation of ethylbenzene in batch culture in different studies

Strain	Maximum substrate concentration (mg/L)	μ_max_ (1/h)	K_s_ (mg/l)	K_i_ (mg/l)	T (°C)	pH	References
Mix culture adapted with petrochemical residue	220	0.2090	37.77	62.62	32	7	This study
*pseudomonas putida f1*	80	0.26	1.5	20	35	7	[36]
*Pseudomonas* species	80	0.13	0.36	-------	30	----	[24]
Aerobic bacterial consortium	100	0.05	0.11	100	30	6.2–6.9	[33]
Immobilized *Pseudomonas putida* and *Pseudomonas fluorescens*	150	0.012	236	429	25	6.4	[23]
*Comamonas sp. JB*	50	0.0064	49.35	-------	30	7.5 to 8.0	[37]

The parameters’ values are for the **Andrews** model if a value of K_i_ is given. If the K_i_ value is not given the parameters’ values are for the **Monod** model

In addition, when the obtained values for styrene and ethylbenzene were assessed, the styrene showed lower K_s_ value than ethylbenzene. Hence, the estimated K_s_ value on styrene indicates that affiliation of mixed culture to styrene is higher than ethylbenzene. Besides, the difference between μ_max_ factors revealed limited styrene degrading capability (in contrast to ethylbenzene) in the environment of an activated sludge plant used for treating petrochemical wastewaters. In addition, different values of μ_max_ demonstrate different pathways in order to completely catabolize the selected components using the microbial species picked to attack and catabolized the carbon sources [39].

The achieved outcome through this part of study revealed that mixed culture has a good resistance to the substrate inhibition compared to other culture studies. Furthermore, the main reason for the differences in biodegradation kinetics of these substrates could be attributed to: a) metabolic pathways which according to former studies leads to the formation of the intermediate products caused by the reaction rate. b) Mass transfer process as an essential part of transforming the substrate into bio-cells, and c) Additional physicochemical conditions that might affect the biodegradation reaction rate [39, 40].

### Dual substrate experiment and substrate interactions

Single substrate kinetic parameters cannot deal with the tough situation detected in biodegradation of the mixed toxic substrates. Thus, experiments with specific conditions were performed to evaluate the effects of interactions among substrates on styrene and ethylbenzene degradations (as a binary mixture) which have been nearly neglected by other authors. Since in low level of substrate concentrations the differences between the Andrews model and the Monod model are negligible and additional parameters in the Andrews model are ineffective on fitting the model to the experimental data, the Monod model was chosen to evaluate the mixture experiments parameters in this section. The results of a biodegradation experiments are shown in Fig. 4. Although the mixed bacterial culture used both substrates at the same time during most of the treatment period, ethylbenzene biodegradation as well as ethylbenzene depletion began before that of styrene.

This specifies that the styrene degradation was inhibited due to the existence of second substrate i.e. ethylbenzene, while the presence of styrene had little effect on ethylbenzene depletion. In addition, the prolonged lag and poor degradation at early times could be related to the competition of microorganism and its adaption to the new situation in order to predominate the special species which can degrade substrates easily. On the other hand, smooth and late biomass growth probably support the construction of the intermediates during the biodegradation reactions.

**Fig. 5:**
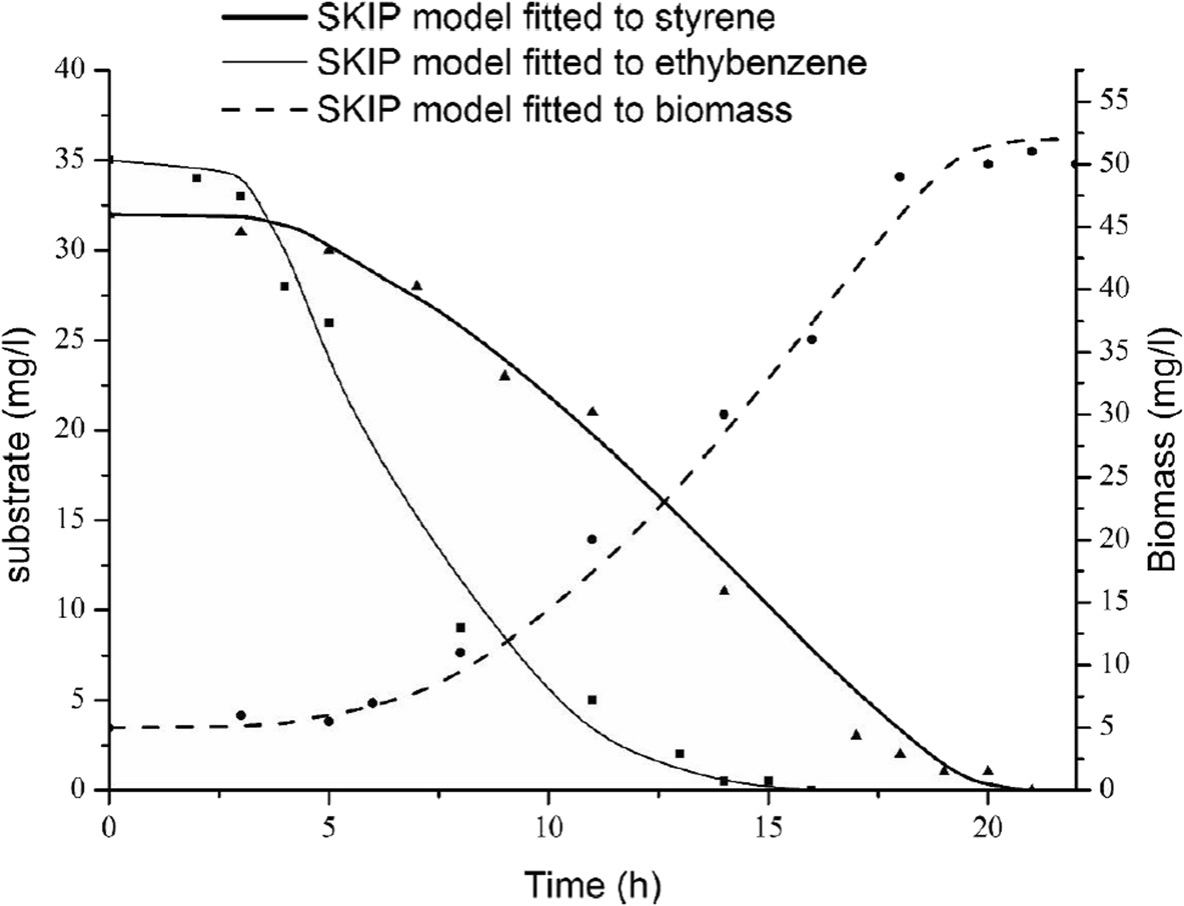
Styrene and ethylbenzene dual degradation and biomass growth experimental data (shapes) and simulation outcomes acquired via SKIP model (lines)

The SKIP model (Eq. 6) was used to define any interactions among the observed substrate. To obtain the values of the interaction parameters, kinetic parameters from single substrate experiments were used and substituted into substrate depletion and biomass growth equations (Eqs. 1 and 2). The results are shown in Table 6. As can be seen in Fig. 5 the fitted SKIP model perfectly explains the biodegradation data for styrene and ethylbenzene binary mixture. Although the biomass prediction depicts slightly higher yield than the experimental data. The large value of I_E,S_ compared to I_S,E_ approves the assertion made from experimental outcomes. It also represents a high degree for the inhibition of ethylbenzene on styrene biodegradation versus the insignificant impact of styrene on ethylbenzene consumption. Moreover, Fig. 5 demonstrates that the SKIP model can be used appropriately to fit unspecified types of inhibition between the two substrates.

**Table 6:** Bio-kinetic interactive parameter estimated for styrene and ethylbenzene dual substrate experiments

Model type	Parameter	Value
SKIP	I_S,E_	0.4
	I_E,S_	1.64
Competitive inhibition	K_s S_/K_s EB_	0.16
	K_s EB_/K_s S_	6.4

To evaluate the similarity of metabolic pathway that was used in the catabolism of both components and to estimate the possible compete for the active site, specific growth rate models similar to the SKIP model were determined.

**Fig. 6:**
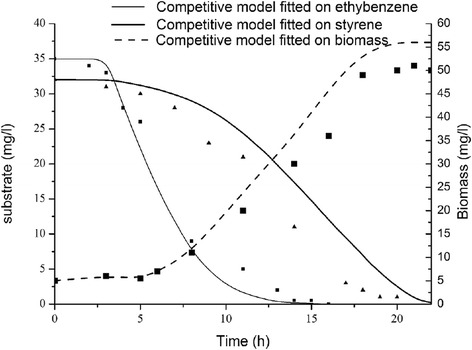
Styrene and ethylbenzene dual degradation and biomass growth experimental data (shapes) and simulation outcomes acquired via purely competitive inhibition model (lines)

The models account for competitive inhibition among dual substrates (Eq. 5). Figure 6 depicts the output curves and the fitness quality of the purely competitive model gained from experimental data. It is obvious that this model does not deliver a correct fit to the experimental data for styrene and ethylbenzene combinations. Potential reasons for inappropriateness of such kinetics and experimental results are interactions among the substrates which are transported into the cytoplasm and enzymatic reaction complexity. Besides, the achieved results suggest that the active site for styrene biodegradation was not similar to ethylbenzene. This theory is supported by the research led by Deebet al. [41] performed for BTEX and MTBE biodegradation. Thus, the inhibition between styrene and ethylbenzene is not thoroughly competitive and due to various biodegradation pathways to metabolize the substrates a single interaction method does not explain the biodegradation kinetics easily. Supplementary studies of the metabolic pathways will make promise to deepen the understanding of interactions between mentioned substrates.

## Conclusion

As outlined above, the biodegradation of styrene and ethylbenzene as a sole and binary source of carbon and energy in water was studied using industrial mixed culture. According to the biodegradation batch experiments and the attained data, it was observed that Andrews model successfully predicted kinetic biodegradation for wide and various ranges of styrene concentration (from 8 up to 220 mg/L) and ethylbenzene concentration (from 12 up to 220 mg/L) in a single substrates experiments. Yet, the simulation obtained from the Monod model in low concentration level for organic component is reliable. The comprehensive biodegradation of styrene and ethylbenzene occurred within various hours after being in contact with the microbial culture. Lag phase in styrene and ethylbenzene biodegradation increased efficiently as the organic concentration upraised. Nevertheless, lag phase time for styrene is slightly higher than that for ethylbenzene and the differences among the two lag phase time were extended at higher concentrations. Microorganism growth as well as organic component depletion for ethylbenzene was slightly faster than that of for styrene. Furthermore, the bio-kinetic factors obtained from single substrate biodegradation studies were used to assess the parameter for the interaction effect. This had a significant effect on biodegradation of a binary mixture. Hence, the styrene and ethylbenzene were used as substrates to evaluate the inhibition of toxic compounds and their interactions in a binary mixture. To the best of our knowledge the interaction between styrene and ethylbenzene has not been investigated until the current study. The outcomes of dual experiments were modeled using a purely competitive model and the SKIP model. Through using the corporate SKIP model, an accurate description for the biodegradation process for a dual mixture of styrene and ethylbenzene was attained. However, the purely competitive model had a poor estimation and fitness for experimental data. Finally, the difference between the biodegradation pathways was the main potential reason for the inaccurate description.

## Footnotes

1 *Web site address:**http://www.tpco.ir/*

2 Web site address: http://www.chem.agilent.com/

## References

[1] Chen W-H, Yang W-B, Yuan C-S, Yang J-C, Zhao Q-L (2013). Influences of aeration and biological treatment on the fates of aromatic VOCs in wastewater treatment processes. Aerosol Air Qual Res.

[2] Ruel SM, Choubert J-M, Budzinski H, Miege C, Esperanza M, Coquery M (2012). Occurrence and fate of relevant substances in wastewater treatment plants regarding Water Framework Directive and future legislations. Water Sci Technol.

[3] Malhautier L, Quijano G, Avezac M, Rocher J, Fanlo JL (2014). Kinetic characterization of toluene biodegradation by Rhodococcus erythropolis: Towards a rationale for microflora enhancement in bioreactors devoted to air treatment. Chem Eng J.

[4] Ahansazan B, Afrashteh H, Ahansazan N, Ahansazan Z (2014). Activated sludge process overview, international journal of environmental science & development 5.

[5] Sannino D, Vaiano V, Ciambelli P (2013). A green route for selective synthesis of styrene from ethylbenzene by means of a photocatalytic system. Res Chem Intermed.

[6] Parreira FV, de Carvalho CR, Cardeal ZL (2002). Evaluation of indoor exposition to benzene, toluene, ethylbenzene, xylene, and styrene by passive sampling with a solid-phase microextraction device. J Chromatogr Sci.

[7] Zhou J, You Y, Bai Z, Hu Y, Zhang J, Zhang N (2011). Health risk assessment of personal inhalation exposure to volatile organic compounds in Tianjin. China Sci Total Environ.

[8] Sofuoglu SC, Aslan G, Inal F, Sofuoglu A (2011). An assessment of indoor air concentrations and health risks of volatile organic compounds in three primary schools. Int J Hyg Environ Health.

[9] US-EPA, List of contaminants and their maximum contaminant level (MCLs). http://water.epa.gov/drink/contaminants/index.cfm. (January 2013).

[10] Liu Y (2007). Overview of some theoretical approaches for derivation of the Monod equation. Appl Microbiol Biotechnol.

[11] De Lucas A, Rodriguez L, Villasenor J, Fernandez F (2005). Biodegradation kinetics of stored wastewater substrates by a mixed microbial culture. Biochem Eng J.

[12] Pala-Ozkok I, Rehman A, Yagci N, Ubay-Cokgor E, Jonas D, Orhon D (2012). Characteristics of mixed microbial culture at different sludge ages: effect on variable kinetics for substrate utilization. Bioresour Technol.

[13] Delhoménie M-C, Nikiema J, Bibeau L, Heitz M (2008). A new method to determine the microbial kinetic parameters in biological air filters. Chem Eng Sci.

[14] Karamanev D, Margaritis A (2006). Kinetic modeling of the biodegradation of the aqueous *p*-xylene in the immobilized soil bioreactor. Biochem Eng J.

[15] Avalos Ramirez A, Bénard S, Giroir-Fendler A, Jones JP, Heitz M (2008). Kinetics of microbial growth and biodegradation of methanol and toluene in biofilters and an analysis of the energetic indicators. J Biotechnol.

[16] Babaee R, Bonakdarpour B, Nasernejad B, Fallah N (2010). Kinetics of styrene biodegradation in synthetic wastewaters using an industrial activated sludge. J Hazard Mater.

[17] Gąszczak A, Bartelmus G, Greń I (2012). Kinetics of styrene biodegradation by Pseudomonas sp. E-93486. Appl Microbiol Biotechnol.

[18] Rene ER, Bernat P, Długoński J, Veiga MC, Kennes C (2012). Use of styrene as sole carbon source by the fungus: optimization and modeling of biodegradation. Pathway Elucidation Cell Membrane Compos Appl Biochem Biotechnol.

[19] Jung I-G, Park C-H (2005). Characteristics of styrene degradation by *Rhodococcus pyridinovorans* isolated from a biofilter. Chemosphere.

[20] Parameswarappa S, Karigar C, Nagenahalli M (2008). Degradation of ethylbenzene by free and immobilized Pseudomonas fluorescens-CS2. Biodegradation.

[21] Kim L-H, Lee S-S (2011). Isolation and characterization of ethylbenzene degrading Pseudomonas putida E41. J Microbiol.

[22] Yeom S-H, Yoo Y-J (2002). Analysis of microbial adaptation at enzyme level for enhancing biodegradation rate of BTX. Korean J Chem Eng.

[23] Shim H, Shin E, Yang S-T (2002). A continuous fibrous-bed bioreactor for BTEX biodegradation by a co-culture of *Pseudomonas putida* and *Pseudomonas fluorescens*. Adv Environ Res.

[24] Littlejohns JV, Daugulis AJ (2008). Kinetics and interactions of BTEX compounds during degradation by a bacterial consortium. Process Biochem.

[25] Abuhamed T, Bayraktar E, Mehmetoğlu T, Mehmetoğlu Ü (2004). Kinetics model for growth of *Pseudomonas putida* F1 during benzene, toluene and phenol biodegradation. Process Biochem.

[26] Reardon KF, Mosteller DC, Bull Rogers JD (2000). Biodegradation kinetics of benzene, toluene, and phenol as single and mixed substrates for Pseudomonas putida F 1. Biotechnol Bioeng.

[27] Shuler ML KF (2002). Bioprocess engineering.

[28] Andrews JF (1968). A mathematical model for the continuous culture of microorganisms utilizing inhibitory substrates. Biotechnol Bioeng.

[29] Okpokwasili G, Nweke C (2006). Microbial growth and substrate utilization kinetics.

[30] Grady C, Smets BF, Barbeau DS (1996). Variability in kinetic parameter estimates: a review of possible causes and a proposed terminology. Water Res.

[31] APHA (1995). Standard methods for the examination of water and wastewater.

[32] Kim D-J, Choi J-W, Choi N-C, Mahendran B, Lee C-E (2005). Modeling of growth kinetics for Pseudomonas spp. during benzene degradation. Appl Microbiol Biotechnol.

[33] Datta A, Philip L, Bhallamudi SM (2014). Modeling the biodegradation kinetics of aromatic and aliphatic volatile pollutant mixture in liquid phase. Chem Eng J.

[34] Lin C-W, Cheng Y-W, Tsai S-L (2007). Multi-substrate biodegradation kinetics of MTBE and BTEX mixtures by Pseudomonas aeruginosa. Process Biochem.

[35] Cox H, Moerman R, Van Baalen S, Van Heiningen W, Doddema H, Harder W (1997). Performance of a styrene‐degrading biofilter containing the yeast Exophiala jeanselmei. Biotechnol Bioeng.

[36] Trigueros DE, Módenes AN, Kroumov AD, Espinoza-Quiñones FR (2010). Modeling of biodegradation process of BTEX compounds: kinetic parameters estimation by using Particle Swarm Global Optimizer. Process Biochem.

[37] Jiang B, Zhou Z, Dong Y, Tao W, Wang B, Jiang J, et al. Biodegradation of benzene, toluene, ethylbenzene, and o-, m-, and p-xylenes by the newly isolated bacterium Comamonas sp. JB, Appl Biochem Biotechnol. 2014;1–9. 10.1007/s12010-015-1671-6 26018344

[38] Chen D-Z, Ding Y-F, Zhou Y-Y, Ye J-X, Chen J-M (2014). Biodegradation kinetics of tetrahydrofuran, benzene, toluene, and ethylbenzene as multi-substrate by pseudomonas oleovorans DT4. Int J Environ Res Public Health.

[39] Smith MR (1991). The biodegradation of aromatic hydrocarbons by bacteria.

[40] Ramos JL, Duque E, Gallegos M-T, Godoy P, Ramos-González MI, Rojas A (2002). Mechanisms of solvent tolerance in gram-negative bacteria. Ann Rev Microbiol.

[41] Deeb RA, Hu H-Y, Hanson JR, Scow KM, Alvarez-Cohen L (2001). Substrate interactions in BTEX and MTBE mixtures by an MTBE-degrading isolate. Environ Sci Technol.

